# Supplementary material for: Adult Onset of Type 2 Familial Hemophagocytic Lymphohistiocytosis After SARS-CoV-2 Vaccination with an Unusual Neurological Onset: The Great Mimic
Source: Diagnostics (Basel). 2025 Nov 26;15(23):3000. doi: 10.3390/diagnostics15233000 (PMC12691091; doi:10.3390/diagnostics15233000)
Supplement: Supplementary file 1 [file diagnostics-15-03000-s001.zip › diagnostics-3883396-supplementary.pdf]

Table S1. The course of the laboratory chemical tests

	LDH (<250 UI/L)	IL2-R (range 158-623 UI/L)	Ferritin (range 12-290 ng/ml)	PCR-EBV
July 2022	130			Not detectable
November 2023	161	2818	964	Not detectable
March 2024			3100	Not detectable
April 2024	122	>7500	902	Not detectable
May 2024	281	822	670	Not done
October 2024	332		146	Not done
November 2024	195	5683	3137	Not detectable
December 2024	183	3552	1881	Not detectable
January 2025	199	4008	898	1989
March 2025	295		1441	4910
